# The Relationship Between Public Interest and Surgical Demand During the COVID-19 Pandemic

**DOI:** 10.7759/cureus.37122

**Published:** 2023-04-04

**Authors:** Maveric K Abella, Terric T Abella, Kyle T Yoshida

**Affiliations:** 1 Department of Surgery, University of Hawaii John A. Burns School of Medicine, Honolulu, USA; 2 Research, University of Hawaii, Honolulu, USA; 3 Department of Mechanical Engineering, Stanford University, Stanford, USA

**Keywords:** elective surgery, orthopaedic surgery, coronavirus, google trends, public interest, covid-19

## Abstract

Introduction: Surgical databases are useful for examining outcomes and case volume to improve care, while public interest data has the potential to track the supply and demand of medical services in specific communities. However, the relationship between public interest data and case volume from surgical databases, specifically during disruptive instances like the coronavirus pandemic, is unknown. Therefore, the purpose of this study is to determine how public interest data is related to the case volume of coronavirus and other surgical procedures performed during the coronavirus pandemic.

Methods: This retrospective study included a review of appendectomy, total hip arthroplasty (THA), and total knee arthroplasty (TKA) cases from the National Surgery Quality Improvement Project and relative search volume (RSV) of hip replacement, knee replacement, appendicitis, and coronavirus from Google Trends from 2019 to 2020. T-tests were used to compare surgical caseload and RSV data before and after the COVID-19 surge in March 2020, while linear models were used to determine relationships between confirmed procedures and relative search volumes.

Results: The RSV for knee replacement (p < 0.001, Cohen's D [d] = -5.01, 95% confidence interval [CI]: -7.64 to -2.34) and hip replacement (p < 0.001, d = -7.22, 95% CI: -10.85 to -3.57) had a large dip during the coronavirus pandemic, while the RSV for appendicitis had a smaller dip (p = 0.003, d = -2.37, 95% CI: -3.93 to -0.74). Linear models showed very strong linear relationships between surgical RSV and surgical volume for TKA (R^2 ^= 0.931) and THA (R^2 ^= 0.940).

Conclusions: There was a significant reduction in the number of elective surgeries, which correlated to drops in public interest during COVID-19. The strong correlations between RSV, surgical volume, and coronavirus cases indicate that public interest can be used to track and predict surgical case volume. Our findings allow for greater insight into the use of public interest data to gauge surgical demand.

## Introduction

Large databases like the American College of Surgeons National Surgical Quality Improvement Program (ACS NSQIP) and the Center for Disease Control (CDC) COVID tracker are useful for examining national caseloads and patient outcomes [1]. However, these large databases often lack more refined geographic and social information that could benefit medical case tracking. Other publicly available tools, such as Google Trends, could be used to fill this gap, allowing for the fusion of large datasets to create more robust, predictive models for enhanced medical case tracking. This could benefit doctors and policymakers alike because it can unveil information useful for recovery from disasters, social inequalities, and medical emergencies like COVID-19, which caused delays in surgeries [2,3]. Additionally, ACS NSQIP provides quarterly, national data with a two-year delay to public access, while Google Trends provides weekly data, divided into specific cities, related to common search interests [4]. Thus, it could also be possible to use both data sources to better understand specific populations and geographies in an expedited manner.

Other studies have used Google Trends to track public interest in surgeries and medical procedures. Mohty et al. used Google Trends to analyze public interest in elective hand surgery [5], while others have used Google Trends public interest in platelet-rich plasma injections [6,7]. Google Trends has also been used to track demand in plastic surgery [8] and compare it to plastic surgery capacity, but not surgical volume [9]. Thus, in the realm of medical research, Google Trends has been used exclusively to provide a measure of public opinion, and its potential to provide additional insight via correlations between RSV and actual surgical volume has not been fully established and is often non-reproducible [10]. Furthermore, most studies dealing with Google Trends track over the course of many years to a decade and are not readily adapted to see details in specific, disruptive instances.

Therefore, in this investigation, we measure the effect of the first COVID-19 peak on the public interest in emergent and elective surgical cases, identify the relationships between the public interest in surgical cases to the public interest in coronavirus and the number of coronavirus cases, identify how emergent and elective surgical case volume changes with public interest before and over the course of the COVID-19 pandemic, and evaluate the feasibility and limitations of using public interest data and national databases for tracking surgical demand and volume.

## Materials and methods

Data acquisition

Google Trends, ACS NSQIP, and the CDC COVID Tracker were queried to measure public interest, surgical case volume, and coronavirus case volume, respectively.

Google Trends

Google Trends was queried to determine the general interest in knee replacement, hip replacement, appendicitis, and coronavirus from January 2019 to December 2021 [4]. Knee replacement and hip replacement are colloquial terms for two common elective procedures, namely, total knee arthroplasty (TKA) and total hip arthroplasty (THA). Joint replacements were chosen because it is a highly known, elective procedure that was expected to decrease during the pandemic due to changes in physical activity and prior literature [1]. Appendicitis was searched as a control because it often results in an appendectomy, the most common emergent surgical procedure on ACS NSQIP whose case and search volume were expected to show minimal shift during the pandemic. Google Trends provides weekly data in units of relative search volume (RSV). This provides a measure from 0 to 100 in the relative search interest for a given search term over a specific time period. Each term was searched individually as was done in previous work with Google Trends since searching multiple terms at once would instead provide a relative value between the searches [5].

American College of Surgeons National Surgical Quality Improvement Program

ACS NSQIP [11,12] was queried to determine the actual caseload of THA (CPT: 27130), TKA (CPT: 27447), and appendectomies (CPT: 44950, 44960, 44970, 44979) during the same time period. Unlike Google Trends data that provide weekly counts, ACS NSQIP provides quarterly counts. ACS NSQIP labels emergent and non-emergent procedures, but both were kept in the dataset since Google Trends data does not similarly differentiate this data. In this dataset, emergent arthroplasty procedures made up less than 1% of the data, while the proportion of non-emergent appendectomy procedures held steady with an average of 44 ± 1.7% over the course of 2019 and 2020. Thus, similar results would be expected if emergent cases were removed or kept for any particular procedure. ACS NSQIP data was filtered for caseloads using R Studio [13] and the Tidyverse package [14]. In total, this study included 120,977 TKA, 80,068 THA, and 86,262 appendectomy procedures from 2019 and 2020.

CDC COVID Tracker

The CDC COVID Tracker provides a daily count of new coronavirus cases beginning from January 2020 [15]. The daily new coronavirus count was downloaded from the tracker to measure the confirmed number of coronavirus cases.

Data analysis

Matlab (Mathworks) and its statistics package were used to plot and analyze the data from Google Trends, ACS NSQIP, and the CDC COVID Tracker.

Changes in Public Interest Before and After the COVID-19 Peak

Raw weekly averages for RSV data were plotted with confirmed case counts to observe the trends over the course of 2019 and 2020. The RSV for knee replacement, hip replacement, appendicitis, and coronavirus during the five weeks preceding and following the March 14 peak in coronavirus RSV were compared using a T-test with a significant threshold of p = 0.05 [5]. The standardized mean difference, Cohen’s D, was calculated to determine the effect of peak coronavirus public interest on itself and the other medical search terms.

Relationship Between Coronavirus and Emergent and Elective Surgical RSVs

Knee replacement, hip replacement, and appendicitis RSVs were plotted against coronavirus RSV and the actual CDC new coronavirus case count. A linear model was fit to the data, and root mean square (RMS) error was calculated [6]. The linear model was compared against a constant model, and the F-statistic and p-value for this comparison were provided. When comparing the CDC data with the weekly RSV data, the sum of the coronavirus cases during that week was used.

Relationship Between Public Interest and Actual Case Count

When comparing the RSV data with the number of cases from ACS NSQIP, quarterly data was used by taking the mean RSV over each quarter in 2019 and 2020. When comparing the RSV data with CDC data, weekly data was used by taking the sum of new coronavirus cases for each week in 2019 and 2020. Similar to the relationships between coronavirus and other surgical RSVs, the best linear model fit was calculated.

## Results

Qualitative observations of surgical and COVID-19 case volume and RSV

**Figure 1:**
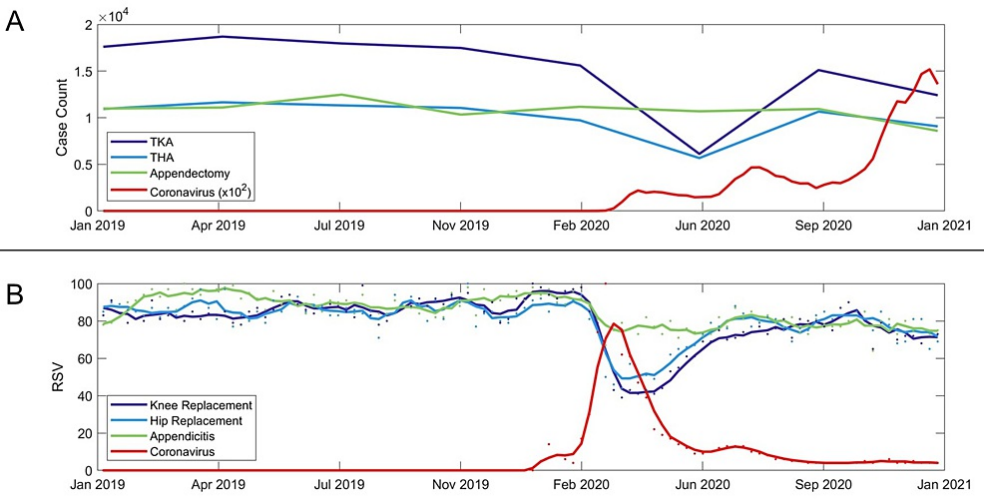
Case count and RSV for coronavirus and surgical terms (A) Case count for total hip arthroplasty (THA), total knee arthroplasty (TKA), appendectomy, and coronavirus from January 2019 to December 2020. Case counts for THA, TKA, and appendectomies are shown quarterly, while coronavirus cases are recorded daily. (B) Relative search volume (RSV) for knee replacement, hip replacement, appendicitis, and coronavirus over the same period. Weekly RSV is indicated with dots, while the monthly moving average is shown with the line.

TKA and THA surgical case volume decreased after the first COVID-19 surge in March 2020 (Figure 1, Panel A). By June 2020 (after the surge), COVID-19 cases continued to increase, while TKA and THA surgical case counts began to recover toward normal volumes. Appendectomy case volume was minimally affected during and after this surge. The changes in TKA, THA, and appendectomy case volume match the RSV trends for knee replacement, hip replacement, and appendicitis, respectively (Figure 1, Panel B). Notably, elective (TKA and THA) surgical volume and the interest in knee replacement and hip replacement fall during the surge and do not fully recover by December 2020. Similar to case appendectomy case counts, appendicitis RSV is minimal by the surge. The peak RSV for coronavirus occurred during the week of March 14, 2020, coinciding with the first coronavirus surge, decreases in the number of surgical cases, and decreases in medical RSV related to those cases. Despite an increase in the number of coronavirus cases through December 2020, the RSV for coronavirus decreases over the same time frame.

Changes in the public interest before and after the COVID-19 RSV peak

**Figure 2:**
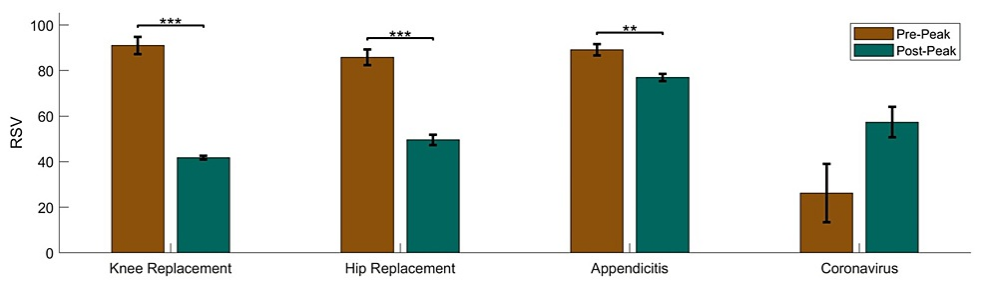
Relative search volume before and after the peak in coronavirus RSV Comparison between relative search volume (RSV) for the five weeks prior (pre-peak) and five weeks after (post-peak) the peak in coronavirus RSV during the week of March 14, 2020. Error bars indicate standard error on the mean. *** indicates p < 0.001; ** indicates p < 0.01; * indicates p < 0.05.

The peak RSV for coronavirus occurred on March 14, 2020. When comparing the five weeks before (pre-peak) and after (post-peak) this peak using a T-test (Figure 2 and Table 1), it was found that there was a decrease in RSV for knee replacement (p < 0.001), hip replacement (p < 0.001), and appendicitis (p = 0.003). There was also an increase in RSV for coronavirus, but this difference was not statistically significant (p = 0.063). There was a very large effect size (Cohen’s D) for all search terms, indicating that the coronavirus RSV surge during the week of March 14, 2020, significantly impacted the RSV for medical procedures. The peak in RSV for coronavirus corresponded with a large, negative effect on RSV for all other RSV search terms, with Cohen’s D ranging from -2.3 to -7.22 for RSV terms related to surgical procedures and a Cohen’s D of 1.23 for coronavirus.

**Table 1:** Changes in RSV before and after the peak in coronavirus RSV The relative search volume (RSV) for colloquial medical terms was compared for the five weeks before (pre-peak) and after (post-peak) the coronavirus RSV peak during the week of March 14, 2020. Mean and standard deviations (SD) are provided pre-peak and post-peak alongside the p-values (p) from the T-test and Cohen’s D (d) with 95% confidence intervals (95% CI).

	Pre-peak	Post-peak		Cohen’s D
	Mean (SD)	Mean (SD)	p	d (95% CI)
Knee replacement RSV	91.0 (8.5)	41.8 (1.8)	<0.001	-7.22 (-10.85, -3.57)
Hip replacement RSV	85.8 (7.7)	49.6 (5.0)	<0.001	-5.01 (-7.64, -2.34)
Appendicitis RSV	89.2 (5.5)	77.0 (3.5)	0.0032	-2.37 (-3.93, -0.74)
Coronavirus RSV	26.2 (28.7)	57.4 (15.0)	0.0632	1.23 (0.06, 2.47)

Relationship between coronavirus and emergent and elective surgical RSVs

**Figure 3:**
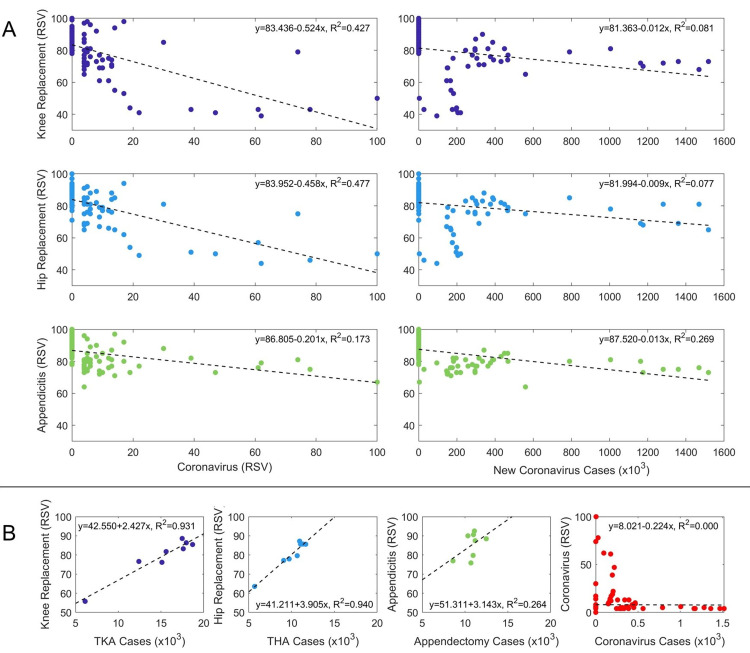
Relative search volume relationships (A) Scatter plots of relative search volume (RSV) for knee replacement, hip replacement, and appendicitis with respect to coronavirus RSV and new coronavirus cases. (B) Scatter plots of knee replacement RSV with respect to total knee arthroplasty (TKA) cases, hip replacement RSV with respect to total hip arthroplasty (THA) cases, appendicitis RSV with respect to appendectomy cases, and coronavirus RSV with respect to coronavirus cases. Equations and R^2^ values for the dashed line describe the best-fit linear model to the data.

As the RSV for coronavirus increases, the RSV for surgical procedures decreases (Figure 3, Panel A; Table 2). Similarly, as the number of coronavirus cases increases, the RSV for surgical procedures decreases. The linear models indicate that knee replacement and hip replacement RSV are moderately correlated to coronavirus RSV (Knee: R^2^ = 0.427, p < 0.001; Hip: R^2^ = 0.477, p < 0.001) and weakly correlated to new coronavirus cases (Knee: R^2^ = 0.081, p = 0.003; Hip: R^2^ = 0.077, p = 0.004). Lastly, appendicitis RSV is moderately correlated with coronavirus RSV (R^2^ = 0.173, p < 0.001) and new coronavirus cases (R^2^ = 0.269, p < 0.001). There were poor linear relationships relating surgical RSV to both coronavirus cases and coronavirus RSV, with R^2^ values all being less than 0.5; however, all of these linear models were statistically significant compared to a constant model.

**Table 2:** Linear model results Linear model results when comparing relative search volume (RSV), coronavirus case counts, and surgical case volume. The RMS error (RMSE) and R^2 ^values for the best linear fit are displayed along with the F-statistic (F) and p-value (p), comparing the linear fit against a constant model.

		Relative Search Volume			
		Knee Replacement	Hip Replacement	Appendicitis	Coronavirus
Coronavirus RSV	R^2^	0.427	0.477	0.173	-
	RMSE	10.600	8.330	7.650	-
	F	76.100	93.200	21.300	-
	p	< .001	< .001	< .001	-
Coronavirus case count	R^2^	0.081	0.077	0.269	0.000
	RMSE	12.400	11.100	7.190	17.400
	F	9.000	8.460	37.500	0.002
	p	0.003	0.004	< .001	0.965
Surgical case count	R^2^	0.931	0.940	0.264	-
	RMSE	2.980	2.080	6.140	-
	F	80.700	93.800	2.160	-
	p	< .001	< .001	0.192	-

Relationship between RSV and actual case count

Knee replacement RSV and hip replacement RSV showed strong linear relationships and correlations to the TKA and THA case counts, respectively (Knee: R^2 ^= 0.931, p < 0.001; Hip: R^2 ^= 0.940, p < 0.001). (Figure 3, Panel B; Table 2). Appendicitis RSV showed a poor relationship and a moderate but insignificant correlation to the appendectomy case counts (Appendectomy: R^2 ^= 0.264, p = 0.193). Coronavirus RSV showed a poor relationship and no correlation to the coronavirus case counts (R^2 ^= 0.000, p = 0.965).

## Discussion

Tracking demand and incidence of medical procedures is important for ensuring adequate access to care throughout the community. Demand and interest in care can fluctuate based on geographic region, time, population, racial disparities, and current events [1]. Our investigation shows that correlations exist between Google Trends data and national databases like ACS NSQIP and CDC COVID-19 Tracker. As national databases like ACS NSQIP do not always have precise and timely geographic information, it could benefit forecasters to use Google Trends data to augment and improve the estimation of changes in medical cases and interest during disruptive events like COVID-19. Furthermore, it is important to understand how information from databases can be interpreted as ChatGPT and other natural language processing tools become more common.

As expected, we found that emergent surgeries showed smaller changes in surgical volume and RSV in comparison to elective procedures before and after the COVID-19 peak in RSV. Notably, there was still a decrease in emergent surgical volume and RSV during the COVID-19 outbreak in March 2020. These reductions may be a result of decreased emergency surgical operations and admissions to hospitals due to fear and anxiety about the coronavirus. Mulita et al. reported that patients in need of emergent surgeries presented with delayed onset of symptoms compared to before the COVID-19 outbreak, which resulted in longer hospital stays and operation durations [16]. These reductions in RSV and surgical volume for emergent surgeries, coupled with existing literature that shows how fear and anxiety from COVID-19 resulted in unnecessary delays in treatment, reflect a dangerous situation with serious consequences for public health.

We also found that there were strong correlations between search volume and surgical volume both before and after the COVID-19 peak for all elective surgical procedures evaluated. This suggests that while elective surgery RSV is primarily used as an indicator of interest, it could also be used to gauge demand, idempotent to the presence of acute, disruptive events. The findings that the drop in demand for elective surgeries after the COVID-19 peak on March 14, 2020, caused significant decreases in search volume [5,17] is supported in this study as we found that the COVID-19 peak increased COVID-19 RSV and decreased elective surgery RSV and surgical volume. By finding the algebraic relationship between RSV and caseloads, models can be generated to accurately predict surgical volume as RSV is provided daily by Google Trends.

Interestingly, after the March 2020 surge, despite a continued increase in coronavirus cases through the end of 2020, public interest and caseloads of surgical procedures recovered (Figure 1), suggesting that COVID-19 case volume alone was not a good predictor of surgical case counts or interest during the COVID-19 pandemic. On the other hand, surgical RSV data had the greatest correlation to surgical case counts, therefore providing the most reliable estimate of surgical volume. Although surgical RSV data had a high correlation to surgical case counts, coronavirus RSV did not share a high correlation to coronavirus case counts (Figure 3, Panel B). Furthermore, as the COVID-19 pandemic continued, there was a growing gap between coronavirus RSV and coronavirus case counts, with RSV decreasing, while coronavirus cases rapidly increased (Figure 1). This large deviation shows a limitation in using public interest data because there may also be times in which public interest may not correspond to actual case volume.

In the future, national medical datasets like ACS NSQIP can be used alongside public interest data like Google Trends for predictive analytics, with approaches utilizing tools like deep learning or Bayesian inference. This system would also be able to adjust and make predictions for acute, disruptive events by integrating disturbances to the model such as public interest data. The ability of Google Trends to be multilayer filtered by geographic region such as city and state [18] allows for cross references with census data to extract racial, political, and socioeconomic information. More interesting analyses on the relationships between medical data, RSV, and census data can be used to understand the demand for medical care in multiple groups of people, thus allowing the medical community to locate and track the changes in global disparities in near real time. In response, research, policy, or educational programs could be created to meet the needs of disparities unique to certain communities [19]. Communities that have a medical need that can be identified through public interest can be the target of the deployment of remote, in-home diagnostic tools and resources [20]. It is understood and accepted that different groups of people are not equally affected by disease and medical treatment. The incorporation of Google Trends data is critical because it provides unprecedented amounts of social and demographic information, which is not provided in traditional medical datasets. The incorporation of Google Trends data can be used to locate the current and future disparities in real time, which will close the gap in providing timely, targeted medical care.

Some limitations of our study include the lack of data from January 2019 to December 2021. However, other studies examine and highlight how some of the public interest in surgery changed before the coronavirus pandemic. Furthermore, there may be omissions from both the ACS NSQIP database and CDC Coronavirus Tracker, which may slightly change some of our findings. Lastly, our search queries were based on terms used in prior studies, but there could be additional or alternate terms in which people are interested that can change throughout an emergent scenario like the COVID-19 pandemic. Because of this, our study does not fully detail every term that could result in changes in caseload or medical interest. For instance, interest in the term "vaccine" may have increased later in 2022, which could have changed the demand for surgeries as it became safer for patients to return to hospitals. However, there would likely be similar results since many of these grouped search terms show strong correlations [21].

## Conclusions

Our investigation showed that strong correlations exist between public interest data from Google Trends and national databases like ACS NSQIP and the CDC COVID-19 Tracker. We also found decreases in RSV and case volume for emergent surgeries during the COVID-19 pandemic, which reflects serious consequences for patients who may have delayed their treatment due to fear and anxiety about the coronavirus. Because of these strong relationships, forecasting and understanding demand stratified by race, time, geography, and more could be done with the fusion of these data streams to evaluate public health. Future work could go into creating specific predictive models to see how geopolitical events change the demand as well as further stratification of the data to assess specific regions or populations.
